# Spatial structure and growth dynamics of fairy rings formed by *Marasmius oreades*

**DOI:** 10.1098/rsos.260575

**Published:** 2026-08-12

**Authors:** Boel Olsson, Markus H. Thorén, Ove Eriksson, Hanna Johannesson

**Affiliations:** ^1^ Department of Ecology, Environment and Plant Sciences, Stockholm University, Stockholm, Sweden; ^2^ Royal Swedish Academy of Sciences, Stockholm, Sweden

**Keywords:** fairy ring, *Marasmius*, soil, metagenomics

## Abstract

Despite the long-term fascination of fairy ring-forming fungi, many aspects of their biology remain unresolved. It is not known why the mycelium grows as an annular structure, rather than a solid disc, or why it grows radially outwards. Different theories have been suggested, but none have been tested experimentally with molecular resolution. Here, we undertook a metagenomic approach to study the growth dynamics of the fairy ring-forming fungus *Marasmius oreades*. We confirmed that *M. oreades* grows as an open ring by detecting its DNA in metagenomes of soil collected along transects from two fairy rings. We showed that the mycelia forming rings are dikaryotic by identifying two mating-type alleles and showing genome-wide heterozygosity. We tested five hypotheses for the mechanism of radial outgrowth using transplantation experiments. The results were most consistent with a transient-escape hypothesis, and suggest that the mycelium avoids inhibitory factors present at the back edge of the mycelial growth front. Our study demonstrates the power of metagenomics for studying fungi, showing that detailed genomic information can be recovered directly from soil. This approach enables the investigation of cryptic aspects of fungal biology, such as the growth of fairy ring-forming fungi, in natural settings that would otherwise remain hidden.

## 1. Introduction

Fungi comprise a highly diverse group of heterotrophs, not only in terms of species but also in terms of morphology. Fungi can be unicellular, like yeast, or multicellular, like mushroom-forming fungi. They can be microscopic or macroscopic, and they can attain a huge size. The largest known fungal individual is of a honey mushroom, *Armillaria ostoyae*, found in Oregon, covering an area of 10 km^2^ [1–3]. However, much of this diversity in growth forms, and what factors determine them, remains to be discovered. In particular, there is a great need for studies addressing the growth characteristics of fungi in their natural environment.

This study aims to uncover the growth patterns behind a phenomenon called ‘fairy rings’. A fungal fairy ring is a mycelial structure and vegetative growth strategy characterized by fruiting bodies organized in the shape of a ring, though other shapes such as arcs and half-circles are also common [4–7]. More than 100 fungal taxa have been observed to form fairy rings [4], and similar growth patterns also occur in clonal plants [8,9]. Fairy rings can be ‘tethered’ to a specific individual tree with which it forms mycorrhizae, as is the case for the basidiomycete *Gomphus clavatus* [10]. Alternatively, the fairy ring grows without any symbiotic interaction with any specific plant species or individual, and grows radially outward over time as it is only constrained by the environment [4,11]. For the latter type, the fungus is likely a decomposer. It is the unbound radially outgrowing type of fairy ring which is the focus of the research here.

Although it has been known since the early 1800s that fungi can form fairy rings [12], the ecology and underlying growth patterns of fairy rings remain unclear. For example, the mycelia forming the fairy rings are expected to die in the centre. However, while it is widely assumed that fairy ring mycelium develops as an annular structure rather than a solid disc, this assumption is based on the ocular observation of mycelia in soil and has so far not been tested with high-resolution molecular techniques. Another key aspect that needs further investigation is the mechanisms behind the formation of fairy rings in fungi, i.e. why they grow radially outwards. Different models have been proposed in order to explain this growth pattern. Fairy ring growth patterns have, for instance, been modelled based on Gray–Scott reaction–diffusion equations to describe how fungal biomass density and resource concentration change over time and space [13,14]. However, field experiments from the early twentieth century have shown that the fairy ring-forming species *Agaricus praerimosus* (syn. *Agaricus tabularis*) and *Clitocybe nebularis* were unable to recolonize the centre, despite having replanted resources vital for growth there [7]. It has also been suggested that the radial outgrowth in woodland fairy rings is caused by ‘intra-mycelial polarity’ that generates a source–sink gradient within the mycelium [15]. This idea has been tested experimentally without clear results [16]. Mathematical simulations have shown that the presence of self-inhibitory factors, such as self-DNA, is necessary for the mycelium to form rings rather than mats [17]. As of yet, there are no conclusive results from empirical data to support these models. More extensive efforts to experimentally test these, and other, models are therefore needed to understand fairy-ring growth dynamics.

In this study, we investigated the growth patterns of the basidiomycete fairy ring-forming fungus *Marasmius oreades.* First, we confirmed that the fungus grows in the form of a hollow circle, and not a solid disc. Second, we confirmed that the mycelia of the investigated rings grew as expected from a typical basidiomycete, harbouring nuclei from both parents in equal frequency and carrying two alleles at the mating-type locus. The first two steps were done by using metagenomic sequencing to investigate fungal DNA in soil cores taken along transects across two fairy rings of *M. oreades*. Subsequently, we used a transplant experiment in which parts of the rings were moved to different places and in different directions across the ring, and used the same molecular technique to screen for fungal growth 14 months after the transplantation. With this approach, we tested five hypotheses to explain fairy ring growth:

— *The continue-forward hypothesis* suggests that hyphae maintain an intrinsic growth polarity. If the mycelial orientation is altered, hyphae will continue to grow in the new direction they are now facing. This growth pattern could be caused by mechanisms such as intra-mycelial polarity [15] and/or the presence of self-inhibitory factors [17].
— *The escape hypothesis* suggests that the fungus grows outwards in order to escape unsuitable conditions in the ring centre. This may be owing to depletion of resources or that the fungus or an organism tied to the fungus may release toxic compounds, hampering growth inwards in the ring. If the fronts of the hyphae are experimentally turned inwards, they would reverse the direction of growth. This hypothesis can be explained by the diffusion–reaction models [13].
— *The transient-escape hypothesis* suggests, similar to the escape hypothesis, that the fungus grows outwards to escape unsuitable conditions, with the difference being that the unfavourable conditions are transient and disappear over time. The unfavourable growth conditions would then be restricted to the area close to the back edge of the mycelial growth front, while the conditions in the ring centre are restored. This hypothesis could be explained by the self-inhibition model [17].
— *The ring-coordination hypothesis* suggests that the radial growth is maintained through ‘intra-mycelial communication’, where cell signalling coordinates the ring shape. If mycelium is transplanted to a different part of the ring, it would adopt the growth direction of its new neighbours to preserve ring integrity. This hypothesis could also be in line with the intra-mycelial model [15].
— *The compass hypothesis* suggests that the ring's growth is oriented as a compass, where hyphae are primed to be oriented towards specific cardinal directions at formation. Accordingly, if the northern section of a ring were moved towards the south, it would still grow northward. This idea is not included in any of the previous models listed above.

These five hypotheses are not mutually exclusive, and more than one hypothesis may be supported by a particular transplant. However, used in combination, the results from the transplant experiment will give indications of the underlying mechanisms behind fairy ring growth.

## 2. Methods

### 2.1. Description of study system: *Marasmius oreades*

**Figure 1.**
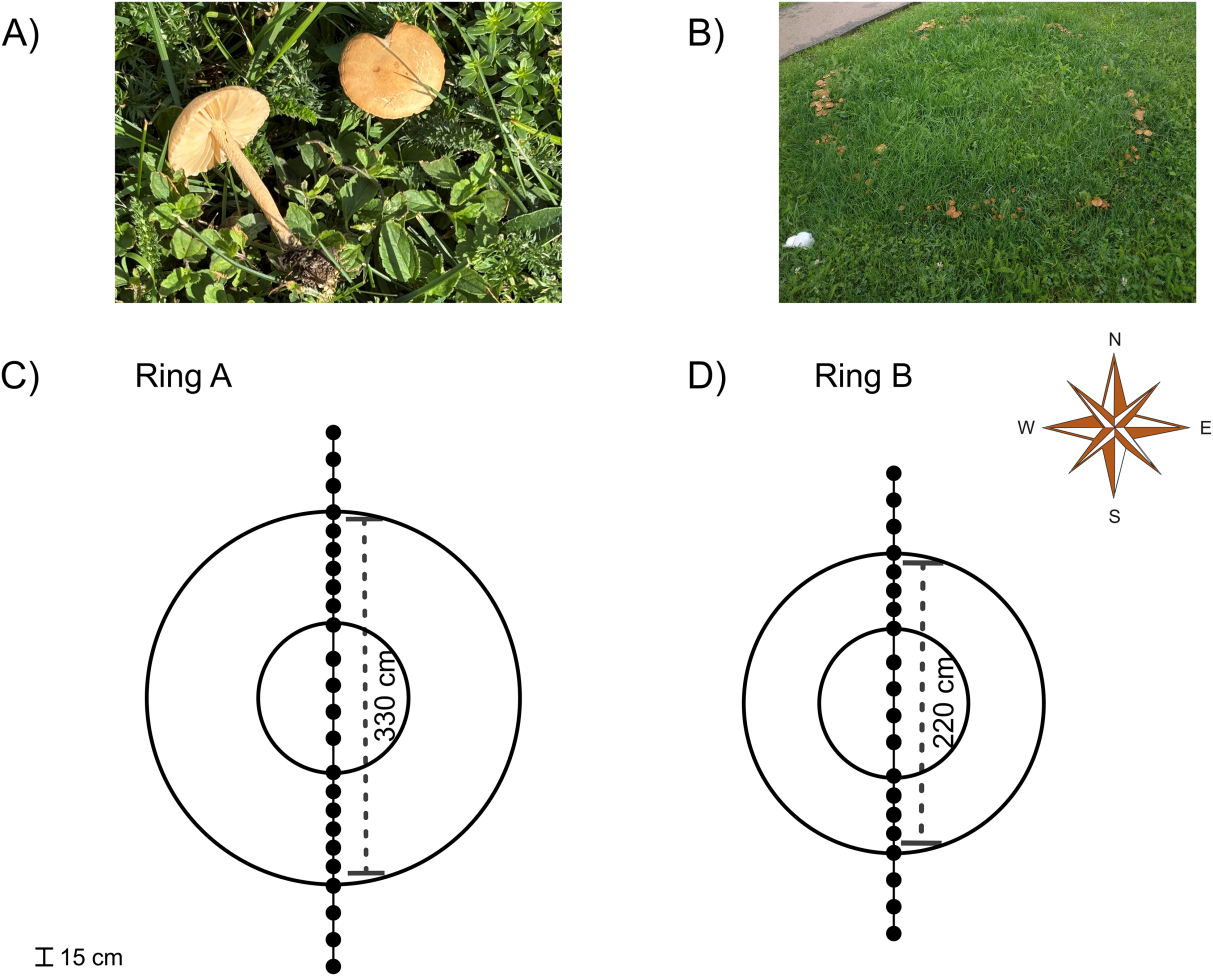
*S*tudy species and overview of soil sampling design for the transect study. *M*arasmius* oreades* (A) fruiting bodies and (B) fairy ring (photos by H.J. and B.O.). The sampling design for (C) ring A and (D) ring B. The visually identified growth front is the area between the two circles of each ring. The transects (solid black lines) were drawn in a north–south direction, with a start and endpoint 60 cm outside of each outer growth front edge, thus covering the outside, growth front and inside of the rings. The spacing between the samples collected from the outside and inside of the ring was about 20 cm, and across the growth front, the spacing was about 15 cm. A total of 24 soil cores (indicated by black dots) were collected from ring A (six from the outside, 14 from the growth fronts and four from the inside). The sampling procedure and design were similar for ring B, but as the diameter and growth front width were smaller, only five samples were collected from each growth front region, leading the total number of samples from ring B to be 20. The rings and distance between sampling points are drawn to scale.

*Marasmius oreades*, also known by the common name ‘the scotch bonnet’, is a basidiomycete in the order Agaricales. Like other agaricomycetes, *M. oreades* forms mushrooms, or fruiting bodies, with special gill-like tissues carrying cells called basidia beneath the cap. It is in the basidia that meiosis occurs and spores are produced. *Marasmius oreades* forms small and pale mushrooms from May to November, and can be found on lawns and meadows all over the Northern Hemisphere (figure 1A,B). Previous research has suggested that each ring is formed by a single mating event between two compatible and haploid mycelia, meaning that one ring corresponds to one genet [5,18,19]. This genet is assumed to be dikaryotic, which means that the nuclei of the parents do not fuse, but instead coexist as separate nuclei over mycelial growth. Gametogenesis and spore formation take place in the basidial cells of the mushroom. The ubiquity of and easy access to *M. oreades* fairy rings make it a suitable model system for this study. Furthermore, the high-quality *M. oreades* reference genome [20] enabled us to screen for *M. oreades* in the metagenomic dataset by read mapping.

All sampling and treatments were done on two rings, denoted ring A and ring B, in Berthåga Cemetery, Uppsala, Sweden (59°51′30″ N and 17°34′40″ E). From their fruiting bodies, the rings were identified as being formed by *M. oreades*. For each of the rings, we visually identified the growth front as the region where the growth of the grass was affected and in which the mushrooms were formed. The growth front was variable within each of the rings, but approximately 60 cm wide, and generally slightly wider in ring A. The approximate diameter, measured from the outer edge of one growth front to the other, of ring A was 330 cm and of ring B 220 cm.

The sampling of the transects and the transplantations were done in September 2023, and the screening of the growth after the transplantations was done in October 2024.

### 2.2. Transect study to examine the fairy ring mycelium shape

In total, 24 samples were collected from ring A and 20 from ring B (figure 1C,D). The soil cores, each 5 cm deep, were collected from just below the grass roots. Soil samples were put in sterile 9 cm Petri dishes and homogenized by mixing with a sterile spoon before being transferred to 50 ml Falcon tubes. The tubes were placed on dry ice immediately after collection and stored at –80°C until DNA extraction.

### 2.3. Transplantation treatments to evaluate growth directionality mechanisms

**Figure 2.**
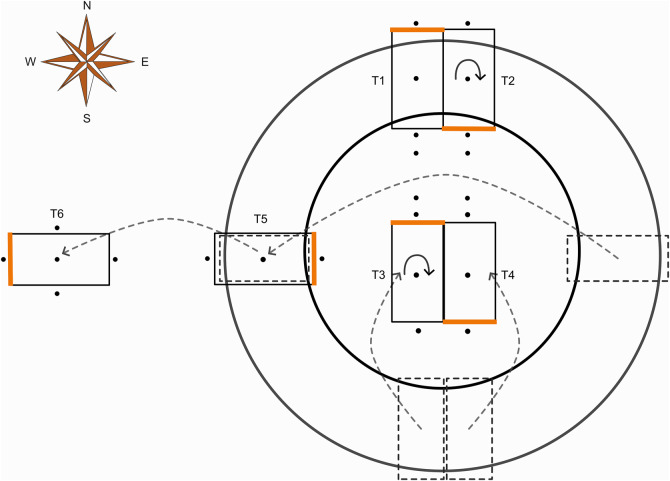
Overview of transplantation treatments and screening to examine the underlying mechanisms for fairy ring growth directionality. Translocations 1–6 (denoted T1–T6) were all done in ring A, but owing to size constraints, T3 and T4 were not done in ring B. Rectangles of size 30 × 70 × 10 cm^3^ (width × length × depth) were moved. The solid black lines show the borders of the rectangular sections that were moved, and the solid light brown lines indicate the side to which the growth direction was at the time of moving. Rectangles with dashed lines show from where growth front sections were dug up if they were moved to different positions in the ring (T3–T6). The transfer of the rectangles is visualized by dashed arrows. Rectangles with dashed grey lines show where we collected soil to cover up the holes that were created when translocating part of the growth front. A semi-circular arrow inside a rectangle means that the section has been turned so that the hyphal tips face a new direction. Presence of mycelia of *M. oreades* was detected 14 months later by collecting 5 cm deep soil cores, which were distributed as indicated by the black dots. Soil cores outside of the growth front section were collected 5 cm from the section edge, except for the extra sampling point between T1 and T3, and T2 and T4. These were collected 25 cm from the edges.

We conducted six transplantation treatments on ring A and four on ring B (figure 2), for which the outcomes together would support alternative hypotheses to explain fairy-ring growth (table 1). Each treatment involved relocating rectangular soil sections (about 30 × 70 × 10 cm^3^: width × length × depth). The rectangle length spanned over both edges of the growth front, meaning that any inhibitory factor that would be present just behind the growth fronts was likely to follow along in the transplantation.

**Table 1.** Expected outcome of mycelial growth under each hypothesis per transplantation treatment.

	hypothesis				
treatment	continue-forward	escape	transient-escape	ring-coordination	compass
T1	continued growth in the original direction	continued growth in the original direction	continued growth in the original direction	continued growth in the original direction	continued growth in the original direction
T2	continued growth in the original direction	no growth at either edge	no growth at either edge	reversed growth northward	reversed growth northward
T3	continued growth in the original direction	no growth at either edge	continued growth in the original direction	no growth at either edge	continued growth in the original direction
T4	continued growth in the original direction	no growth at either edge	continued growth in the original direction	no growth at either edge	reversed growth southward
T5	continued growth in the original direction	no growth at either edge	no growth at either edge	reversed growth westward	continued growth in the original direction
T6	continued growth in the original direction	growth in all directions except backwards	growth in all directions except backwards	no growth	continued growth in the original direction

In the first treatment (T1, figure 2), a rectangle was dug up and placed back in its original position and orientation. This treatment is primarily a control for the effect of disturbing the mycelial growth with our method of transplantation.

In the second treatment (T2, figure 2), a rectangle was dug up and turned so that the presumed hyphal tips faced inward. This treatment primarily tested the *continue-forward hypothesis* (table 1).

In the third and fourth treatments (T3 and T4, figure 2), growth front rectangles were moved to the centre of the ring, one with reversed hyphal direction (T3, figure 2) and another maintaining its original orientation (T4, figure 2). These treatments assessed the environmental conditions within the ring, and hence primarily tested the *escape hypothesis* and the *transient-escape hypothesis* (table 1). These treatments were only performed on ring A owing to space constraints in ring B.

In the fifth treatment (T5, figure 2), a rectangle was relocated from the eastern to the western side of the ring without altering the direction of the hyphal tips (still facing east). This treatment primarily tested the *compass hypothesis* (table 1).

In the sixth treatment (T6, figure 2), a growth front rectangle was moved outside the ring to determine whether ring communication is necessary, primarily testing the *ring-coordination hypothesis* (table 1).

Although each of the treatments focused on testing one particular hypothesis, the results of all treatments also have bearing on the other hypotheses. Table 1 summarizes the expected outcome of the treatments based on each of the hypotheses.

All transplantations took place on 14 September 2023. To track the mycelial growth after the transplantation treatments, we collected soil cores on 15 November 2024 (see figure 2 for placement) using the same sampling and sequencing method as for the transect study. For ring A, all transplanted rectangles could be relocated at the time of sampling. By contrast, the exact locations of the transplants of treatment T1, T2 and T5 in ring B could not be accurately determined, so we only report results from treatment T6 from this ring.

Four soil cores were collected from rectangles from the T1 and T2 transplantations, with one extra sampling point 25 cm from the rectangle edge of the short side facing the other treatment (figure 2). Three cores were collected from rectangles T3–T5, one from the centre and one from each short side, 5 cm from the edge (figure 2). Five cores were collected from T6, one from the centre and one from each side, again 5 cm from the edges (figure 2), to control whether mycelium would start growing in all directions if placed outside of the ring. After collection, the soil samples were stored at –20°C until DNA extraction.

### 2.4. DNA extraction and sequencing

For both the transect and translocation experiments, DNA was extracted from soil using a DNeasy PowerSoil Pro extraction kit, following the manufacturer's protocol (QIAGEN Inc. [21]). The amount of soil that was used for extraction varied from 90 to 230 mg, but the aim was around 110 to 150 mg, in order to extract the amount of DNA according to the sequencing facility's requirements. The DNA was then sequenced with Novogene's PCR-free shotgun metagenomic sequencing service. Novogene performed the library preparation prior to sequencing. The samples were sequenced on two lanes on the NovaSeq X Plus Series machine with 150 bp paired-end reads, aiming for 15G of raw sequencing data per sample.

### 2.5. Bioinformatic analysis

To screen for the presence of *M. oreades* DNA along the fairy ring transects, the raw reads from the sequencing were trimmed from adapters with Trim Galore v0.6.10 [22], and then mapped against the reference genome (NCBI accession number: GCF_018924745.1) using BBMap [23], part of BBTools version 39.19, with default settings. In downstream bioinformatic analyses, the read fractions were normalized by the total number of reads in the sample. The proportion of reads mapping to *M. oreades*, relative to those retained after adapter trimming, served as a measure of *M. oreades* DNA content. The same screening approach was used to detect growth in the translocation treatments, with the addition of removing polyG sequences that were over-represented using fastp v1.0.1 [24] prior to mapping.

From the transect data, we used two approaches to infer whether *M. oreades* mycelia of each ring grew as dikaryons [5,6,25]. First, we called variants in the samples with FreeBayes v.1.3.10 [26] and plotted the genome-wide allelic depths for heterozygous sites in windows of 1 Mbp in 90 kb steps. In this analysis, a mean value around 0.5 was expected for a dikaryotic sample, assuming equal nuclear ratios. Second, we investigated the mating-type locus of each ring. For dikaryosis resulting from a mating event, two divergent alleles at the mating-type locus were expected per ring. *Marasmius oreades* has a bipolar mating system where the *HD* locus (with two *HD* genes: *HD1* and *HD2*) acts as the mating-type determinant [25]. Since the high sequence variation at the *HD* locus may prevent effective read mapping of both alleles, we generated metagenomic assemblies from one sample for each ring (supplementary material, table S1) with SPAdes v1.4.0 [27], and then aligned the published protein sequences of HD1 and HD2 (accession numbers XP_043016060.1 and XP_043016059.1) against the assemblies with tBLASTn, with an e-value threshold of 10^–80^. Identified contigs, carrying putative *HD* genes, were extracted and aligned against the sequences of previously identified *HD* alleles from six other fairy rings [25]. Identified contigs bearing putative *HD* genes were joined into full *HD* haplotypes based on paired-end information and identity to previously identified haplotypes. The resulting nucleotide sequences per ring were aligned with the previously identified *HD* alleles with MAFFT v. 7.450 [28] within Geneious (www.geneious.com). A maximum likelihood tree was generated using IQ-TREE2 v. 3.0.1 [29], where ModelFinder [30] selected HKY+I+R2 as the best fitting model of sequence evolution.

## 3. Results

### 3.1. Sequencing statistics

For the transect part of the study, we yielded 8.93 Gb of raw data on average for all samples, corresponding to 59.95 million reads on average. The average Q30 score for all samples was 89.93%, and the average GC content was 63.22%.

For the transplant experiments, we ended up with a dataset with 51 Gb of raw data in total, corresponding to a total of 3.4 billion reads. On average, there were 1.7 Gb of data and 11.9 million reads per sample. The Q30 score was on average 95.1%, and the GC content was on average 63.5%.

### 3.2. Molecular verification of *Marasmius oreades* mycelium forming annular structures

**Figure 3.**
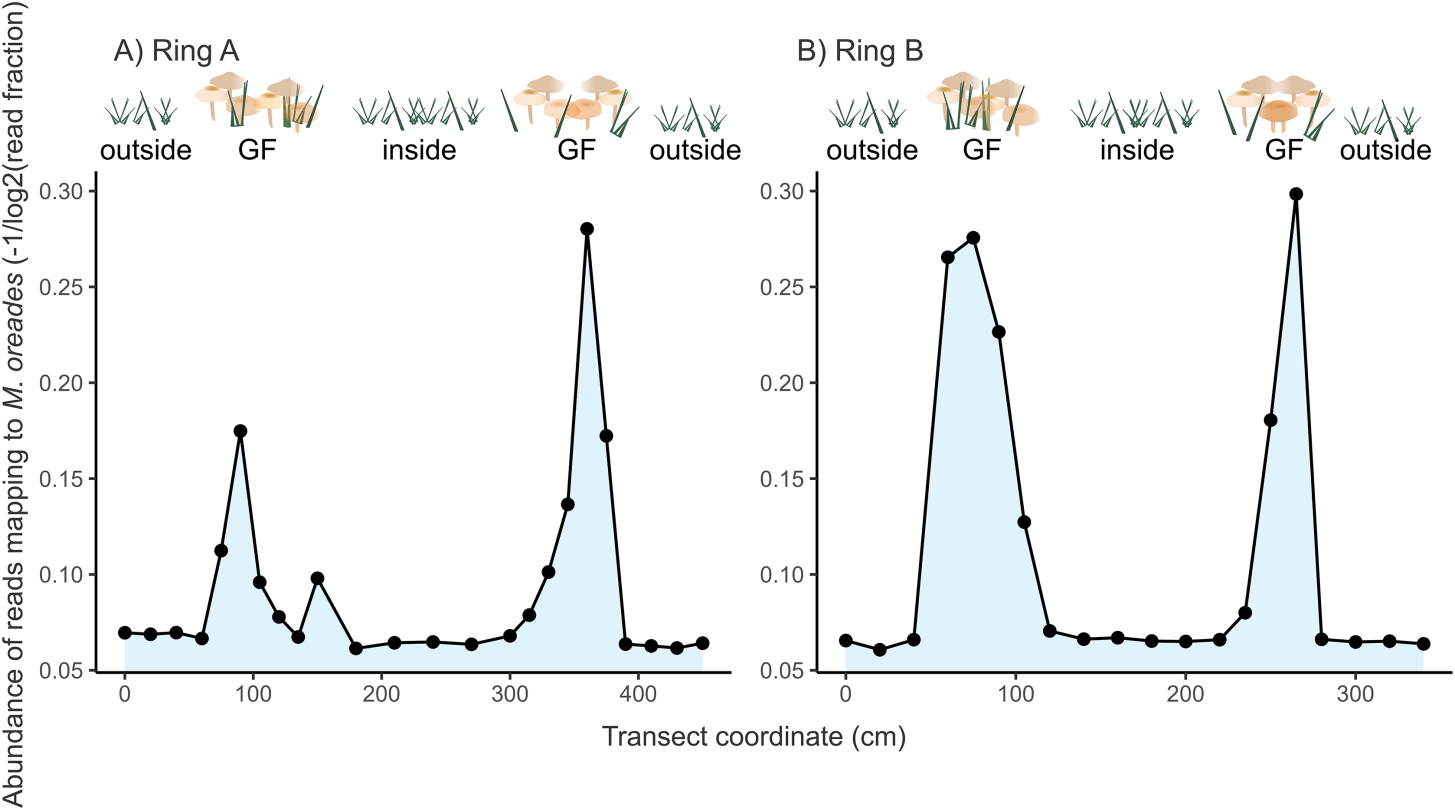
Proportions of reads mapping to the *M. oreades* reference genome along the transects in ring A (A) and ring B (B). Transect coordinates are given on the *x*-axis, and zone type (outside, GF = growth front, inside) is marked above the chart with text and illustration. Black solid lines between the data points are added to visualize changes along the transect more clearly. The *y*-axis shows the abundance of reads within each sample that map to the *M. oreades* reference genome. The fraction is the number of *M. oreades* reads divided by the total number of reads in each sample, and then transformed with –1/log_2_ to make fold-changes between samples easier to interpret. A transformed value of 0.1 is equivalent to 0.1% of the total DNA.

The proportion of *M. oreades* reads was elevated within the growth front regions in comparison with the outside and inside of the ring for both rings (figure 3A,B; supplementary material, table S1). The proportion of *M. oreades* reads was highest at the outer part of the growth front. The proportions of *M. oreades* reads outside and inside the ring were similar, indicating an absence of *M. oreades* DNA in soil inside the ring.

### 3.3. Verification of dikaryotic growth of sampled *Marasmius oreades* fairy rings

**Figure 4.**
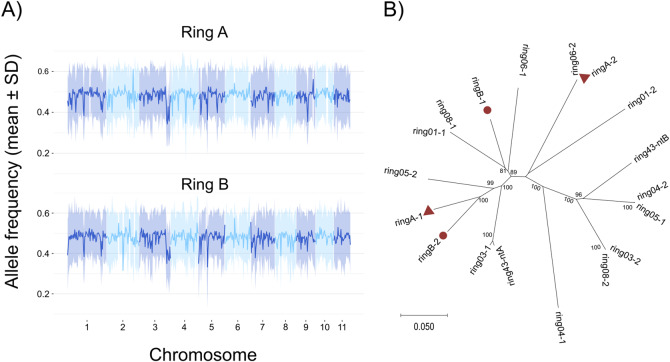
Mean allele frequency along the genomes of the rings and the phylogenetic relationship between mating type haplotypes. (A) The mean allele frequency (*y*-axis, ranging from 0.1 to 0.7) and standard deviation (shaded area) in 1 Mb windows in 90 kb steps of SNPs at biallelic sites detected with FreeBayes along the genome of rings A (upper row) and B (lower row). The chromosomes are ordered in descending size, and the colour of the allele frequencies alternates between dark and light blue to visually differentiate the chromosomes. (B) Maximum likelihood tree of the concatenated nucleotide sequence of *HD1* and *HD2* in ring A (marked with triangles), ring B (marked with circles) and previously identified alleles [25]. The branch length is proportional to the number of substitutions per site, as indicated by the scale bar. The numbers at the nodes represent ultrafast bootstrap support values.

The mean frequency of allelic variants in 1 Mb windows oscillated around 0.5 across the entire genome in both rings (figure 4A). Furthermore, we identified two *HD1* and *HD2* gene variants on separate contigs. For ring A, the sequence of one of the alleles (ringA-2, figure 4B) was identical to a previously identified allele, while the other alleles (ringA-1, ringB-1 and ringB-2) were clearly distinct from all previously identified alleles of the *HD* locus in *M. oreades* (figure 4B). Taken together, these results show that the underground mycelia forming the rings are composed of dikaryotic hyphae and are consistent with the expectations of the vegetative stage of the *M. oreades* life cycle resulting from a mating event of two compatible monokaryons [5,18,19].

### 3.4. Results of the transplantation experiment

**Figure 5.**
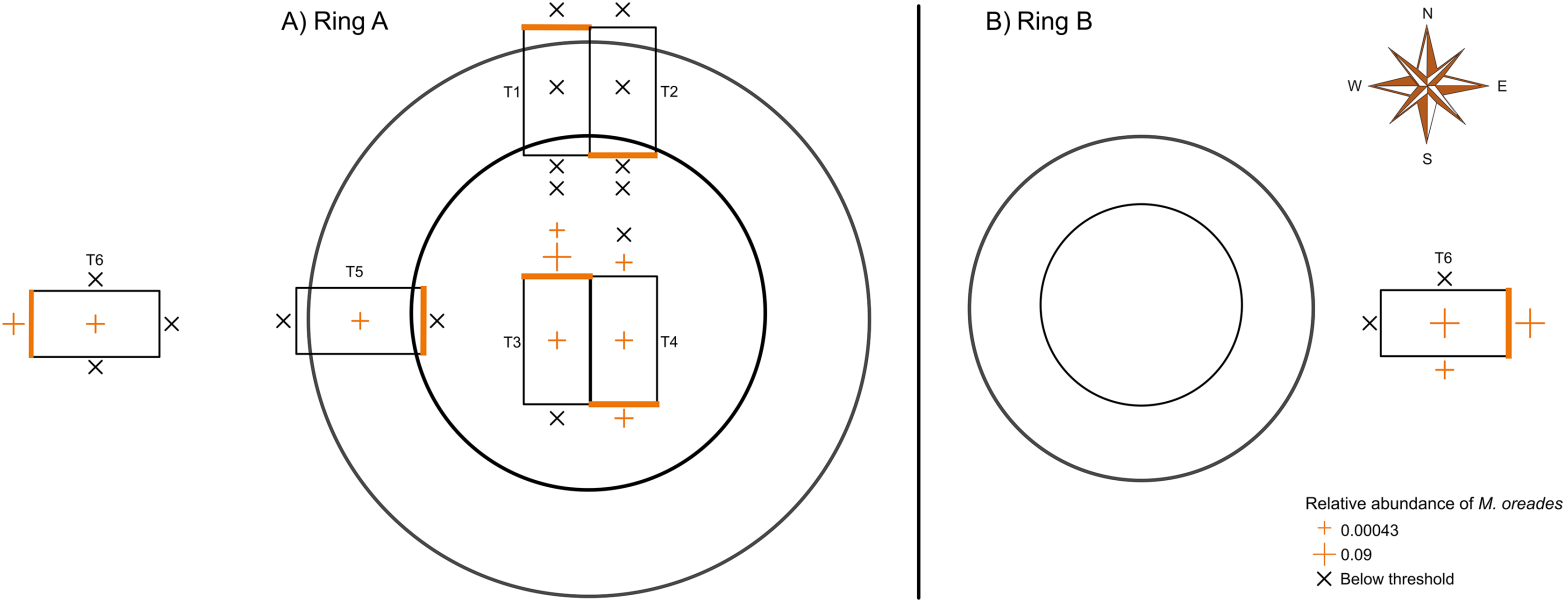
Results from the growth screens of the transplantation treatments of ring A (A) and ring B (B). Treatment denotations (T1–T6) and ring boundaries are indicated as in figure 2. Note that T6 was moved westward in ring A, but eastward in ring B owing to topological constraints in the environment. Presence of *M. oreades* was defined using a threshold of 0.1 on the transformed read fraction (−1/log_2_). For samples above this threshold, plus-sign size is proportional to the raw fraction of *M. oreades* reads (power-scaled for visualization), while samples below the threshold are indicated by crosses of uniform size. Distances between sampling points are not drawn to scale.

The results from the transplantation experiment are shown in figure 5, which summarizes the proportion of *M. oreades* reads from the samples taken 14 months after the transplantation. The complete result from this experiment is available in table S2 in the supplementary material.

In T1, we detected no growth in either direction or in the rectangle centre (figure 5). As this was our control for the effect of disturbing the mycelial growth by transplantation, the result suggests that digging up the rectangle in itself had a negative effect on the mycelial growth. However, note that no growth was detected in T2 as well, which was located very close to T1, while in the other parts of the ring, mycelia were found to grow at least in the centre of the rectangle (figure 5). Taken together, the absence of growth after transplanting in T1 and T2 suggests that mycelium in this part of the ring was less viable than the other parts, rather than that the digging disrupted mycelial growth. Hence, we find T1 and T2 inconclusive for testing the hypotheses outlined in table 1.

For the treatments where transplants were taken from the growth front and moved into the centre of the ring (T3 and T4, figure 2), we concluded the following: first, for T3, which was reversed from its original direction of growth, *M. oreades* continued to grow forward (figure 5). By this observation, the results of T3 do not support the escape, the ring-coordination or the compass hypotheses, but are in line with the expected outcome under both the continue-forward and transient-escape hypotheses. Second, in T4, which was not reversed, the mycelium grew both forward and backwards, which does not correspond to any expected outcome of any hypothesis. However, the result of T4 speaks strongly against the escape and ring-coordination hypotheses, which both predict no growth at all for this transplant (table 1).

The growth pattern in T5 is in line with our expectations for the escape and transient-escape hypotheses, as we detected no mycelial growth at either the forward or back edge of the rectangle (figure 5). This treatment did therefore not support the continue-forward, ring-coordination or the compass hypotheses (table 1).

The mycelium of the rectangle placed outside of ring A (T6, figure 2) continued in its original direction (figure 5A), supportive of the continue-forward and transient-escape hypotheses and rejecting the ring-coordination hypothesis. The same treatment in ring B yielded the same result, with the addition of growth in a southward direction (T6, figure 5B). This growth pattern only fits with the predictions for the transient-escape hypothesis.

Taken together, the transplantation results are most consistent with the transient-escape hypothesis, which was supported in four cases (treatments T3, T5 and both T6). The next most supported hypothesis was the continue-forward hypothesis, which was supported in two cases (T3 and T6 in ring A), but in both these cases, the results were also in line with the transient-escape hypothesis.

## 4. Discussion

The circular pattern of mushrooms, or altered vegetation caused by an underground fungal network, has long puzzled people. A distinctive feature of fairy rings is the regular arrangement of their growth, originating from a single point and spreading outwards. However, many fundamental features of the fairy rings, and what factors govern the typical growth patterns, have remained obscure. Here, we used molecular methods to confirm the absence of mycelia in the centre of the ring, and hence, we verified that the mycelial colony develops as an open circle, rather than a solid disc. Our method is based on metagenomic information, obtained by sequencing DNA directly from soil, and comparing the reads with those of the *M. oreades* reference genome to identify reads from this species along a transect crossing the ring. By this method, we revealed a clear pattern of *M. oreades* DNA being present in the growth front of the fairy ring, but inside the ring, the fungus was detected at very low levels, i.e. in similar levels as outside. Note that, even if the peaks in abundance of *M. oreades* in the growth fronts are clear, especially in the outer part of the growth fronts, the relative amount of *M. oreades* DNA was low. On average, *M. oreades* comprised only approximately 2% of the total DNA within the growth fronts. This low fraction reflects the complexity of soil DNA pools, which potentially leads to extraction and sequencing biases, as well as divergence from the reference genome, and the limited completeness of fungal genomic reference databases. However, the fact that such a small signal was detected, highlights the sensitivity and high resolution of metagenomic sequencing and underscores its value for future in-depth studies of fungal biology directly in their natural environments.

We were also able to use the metagenomic data to study nuclear variation within the mycelia of *M. oreades*, and thereby verify some of the expectations of the *M. oreades* life cycle. Our study is novel in using a metagenomic approach for the purpose of investigating dikaryosis in soil-derived fungi. Our data on heterozygous sites and variation at the mating-type locus show support for the mycelium growing as a dikaryon carrying nuclei of two different mating types in equal frequencies, as previously suggested based on studies of the mushrooms and the released basidiospores [18,25].

Our results from the transplantation are most consistent with the transient-escape hypothesis. Rather than reflecting a permanent change, the observed patterns suggest the presence of a temporary inhibitory factor behind the advancing growth front. The reduced detection of *M. oreades* behind the former front edge, combined with continued forward growth in several treatments, is consistent with the idea that inhibition is spatially structured but not irreversible. The fact that mycelial growth resumed in centre-transplanted treatments further indicates that this inhibitory effect diminishes over time, allowing recolonization in the centre once the growth conditions become favourable again. This temporal dynamic allows us to reject the escape hypothesis, which predicts a more permanent inhibitory mechanism.

The absence of detectable growth forward in T1 and T2, located close to each other at the northern part of ring A, indicates local mycelial death or reduced viability. Such spatial heterogeneity in growth is consistent with previous observations that growth rates can vary substantially around fairy rings [4]. Together, these findings suggest that fairy ring expansion is governed not by fixed exclusion zones but by dynamic and time-dependent interactions within the soil environment. Note that, because several treatments were inconclusive and the experiment was replicated across only two rings, these results should be interpreted as qualitative and preliminary rather than as a formal test of alternative mechanisms. Future studies that collect data from a higher number of rings are needed in order to make any statistical inferences on growth mechanisms behind the fairy rings.

Our tentative conclusion from our data is that the *M. oreades* mycelium grows forward to avoid some unfavourable environment at the immediate back of the mycelial growth front. However, we can only speculate on the factors that make this zone unfavourable. Both nutrient deficiency and toxin release from the fungus are possible reasons and have also been suggested previously [4,14,15]. Another possibility is that the fungus releases self-inhibitory compounds, such as self-DNA [17]. As of yet, there is no chemical evidence to support that *M. oreades* or any other fairy ring-forming fungus produces self-DNA in order to form a ring-shaped mycelium. Whatever the underlying mechanism is, this question needs to be systematically addressed in future studies.

There are several plausible advantages for the mycelium to grow forward as a ring, suggesting that the growth pattern is a result of natural selection. One could argue that it would make more sense for the fairy ring to be dynamic in its growth and exclusively grow towards nutrient-dense areas. However, this kind of sensory-driven growth is probably energetically demanding. Alternatively, the lawns are nutrient-dense everywhere, meaning that there is no selective advantage to grow specifically towards a nutrient stimulus. This hypothesis could be tested by comparing nutrient concentrations at different parts of fairy rings with uneven growth. One should keep in mind, though, that uniform grass lawns are fairly new environments, meaning that the observed behaviour could in fact be an adaptation to more heterogeneous environments.

If we consider the mycelium forming an annular structure, this could be an adaptation to save energy and avoid pathogen invasion, while maximizing the area where resources are exploited. Fungi are known to transport cytoplasm and nutrients from older parts of the colony to support growth and fruiting at the mycelium front [31]. Hence, the fungus is presented with three options for the middle mycelium: actively maintaining it, passively leaving it behind or actively disintegrating it. As illustrated by field observations [7] and our metagenomic results, ring-forming fungi do not maintain the middle mycelium. The middle part of the ring is reasonably less nutrient-dense than the outside since it has already been colonized by the fungus, and there is probably little to gain by maintaining the mycelium there. Translocating available nutrients from the middle and leaving it behind may be less energetically costly than active disintegration. However, active disintegration enables nutrient recycling and allocation to the periphery of the mycelium, in addition to reducing the risk of invasion or predation by viruses, prokaryotes, nematodes and/or other fungi. Though fungi are known to defend themselves chemically against, for instance, nematodes [32–34], it is not unreasonable that by disintegrating the central mycelia, the fungus acts to defend itself by reducing the potential target area for a predator.

## 5. Conclusion

The use of molecular data has fundamentally transformed mycology, revolutionizing how we understand fungal taxonomy, evolution, ecology and population structure [35]. The most common method for taxon identification is metabarcoding or metagenomic analysis that primarily targets the bacterial community. Our approach to search for reads of a specific fungus from metagenomic sequencing of DNA extracted from soil provided unprecedented resolution, and allowed us to infer both presence and intraorganismal diversity of *M. oreades* forming the fairy rings, as it grew in its natural environment.

Considering that the majority of *M. oreades* life cycle takes place in the soil, having detailed information from soil samples is crucial for understanding its adaptations and ecological impact. Though additional studies with data from more rings, complemented with laboratory experiments, are needed to unveil the causal mechanism behind the observed patterns here, we show that it is possible to gain detailed information about fungal genomes directly from soil samples, which enables us to study vegetative growth of fungi in their natural environment. This will provide new insight and ignite new questions regarding the processes behind the fairy ring structure and growth pattern.

## Supplementary Material



## Data accessibility

The sequencing data for this study have been deposited in the European Nucleotide Archive (ENA) at EMBL-EBI under accession number PRJEB108585 [36].
