# Supplementary material for: Spatial structure and growth dynamics of fairy rings formed by Marasmius oreades
Source: R Soc Open Sci. 2026 Aug 12;13(8):rsos260575. doi: 10.1098/rsos.260575 (PMC13483757; doi:10.1098/rsos.260575)
Supplement: Supplementary file 1 [file rsos.260575.zip › rsos.260575/rsos260575_si_003.docx]

Figure S1. Here the transect sampling design is deptictied for as in Fig. 1 C-D, with the addition of the sample index found in the supplementary table right of the sample.

Fig. S2. Data and axes are the same as in Fig. 3, with the addition of the sample index from Fig. S1 next to the data points.
